# Amadori rearrangement products as potential biomarkers for inborn errors of amino-acid metabolism

**DOI:** 10.1038/s42003-021-01909-5

**Published:** 2021-03-19

**Authors:** Rianne E. van Outersterp, Sam J. Moons, Udo F. H. Engelke, Herman Bentlage, Tessa M. A. Peters, Arno van Rooij, Marleen C. D. G. Huigen, Siebolt de Boer, Ed van der Heeft, Leo A. J. Kluijtmans, Clara D. M. van Karnebeek, Ron A. Wevers, Giel Berden, Jos Oomens, Thomas J. Boltje, Karlien L. M. Coene, Jonathan Martens

**Affiliations:** 1 Radboud University, Institute for Molecules and Materials, FELIX Laboratory, Toernooiveld 7, Nijmegen, the Netherlands; 2 Radboud University, Institute for Molecules and Materials, Synthetic Organic Chemistry, Nijmegen, the Netherlands; 3 Department of Laboratory Medicine, Translational Metabolic Laboratory, Radboud University Medical Center, Nijmegen, the Netherlands; 4 Department of Pediatrics, Radboud Center for Mitochondrial Medicine, Radboud University Medical Center, Nijmegen, the Netherlands; 5 van’t Hoff Institute for Molecular Sciences, University of Amsterdam, Amsterdam, the Netherlands

**Keywords:** Metabolomics, Biomarkers

## Abstract

The identification of disease biomarkers plays a crucial role in developing diagnostic strategies for inborn errors of metabolism and understanding their pathophysiology. A primary metabolite that accumulates in the inborn error phenylketonuria is phenylalanine, however its levels do not always directly correlate with clinical outcomes. Here we combine infrared ion spectroscopy and NMR spectroscopy to identify the Phe-glucose Amadori rearrangement product as a biomarker for phenylketonuria. Additionally, we find analogous amino acid-glucose metabolites formed in the body fluids of patients accumulating methionine, lysine, proline and citrulline. Amadori rearrangement products are well-known intermediates in the formation of advanced glycation end-products and have been associated with the pathophysiology of diabetes mellitus and ageing, but are now shown to also form under conditions of aminoacidemia. They represent a general class of metabolites for inborn errors of amino acid metabolism that show potential as biomarkers and may provide further insight in disease pathophysiology.

## Abstract (web summary)

Rianne van Outersterp et al. combine mass spectrometry, NMR, and infrared ion spectroscopy to identify amino acid-hexose conjugates in the blood plasma from patients with metabolic disorders such as phenylketonuria (PKU). These conjugates, or Amadori rearrangement products, are generally not detectable in blood samples from unaffected individuals, and may therefore represent disease biomarkers.

## Introduction

Phenylketonuria (PKU; OMIM 261600) is an inborn error of metabolism (IEM) caused by a deficiency of the phenylalanine hydroxylase (PAH) enzyme responsible for the phenylalanine (Phe) to tyrosine (Tyr) conversion^1^. In untreated patients, this results in elevated Phe levels in body fluids and a neurologically damaging concentration of Phe in the brain that leads to major health issues, including severe cognitive impairment. Also inherited defects in pterin metabolism, leading to a deficiency in the PAH co-factor tetrahydrobiopterin (BH_4_), can lead to pathological accumulation of Phe^1^. The common and well-established treatment strategy for managing Phe levels involves dietary protein restriction that limits Phe-intake. Add-on therapeutic approaches are in use with the supplementation of BH_4_ (Kuvan) to allow for optimal use of (remaining) PAH enzyme activity. For most patients, this strategy leads to positive clinical outcomes^2,3^. Diagnosis and evaluation of treatment efficiency are now largely based on the level of Phe in combination with the Phe:Tyr ratio in either blood plasma or dried blood spots^3^. However, the severity of symptoms is not always directly related to plasma Phe levels^4,5^. This gives a need for additional metabolic markers for PKU that give a better prediction of the clinical outcome and offer a biochemical explanation for the variability in clinical outcome, observed among treated PKU patients.

Over the last decade, untargeted metabolic profiling techniques that evaluate the levels of many metabolites at the same time have emerged as a powerful tool to detect novel metabolites that can serve as functional biomarkers for IEMs^6,7^. Approaches based on liquid chromatography-mass spectrometry (LC-MS) play a key role owing to their high sensitivity and allow the routine detection of metabolites down to nanomolar levels. However, a known challenge related to the use of untargeted LC-MS is the identification of the detected signals (i.e., assigning a full molecular structure to features having a unique retention time and mass-over-charge ratio, *m/z*) as there are often numerous isobaric candidate structures that must be distinguished. A common method used to assist identification is fragmentation MS (MS/MS); however, this approach relies heavily on the completeness of MS/MS libraries and the availability of reference standards, because ion fragmentation patterns remain difficult to reliably predict in silico^8,9^. Furthermore, a more fundamental limitation is that closely related metabolites often display very similar fragmentation patterns. Thus, although untargeted LC-MS experiments are able to reach into previously unexplored parts of the metabolome, a vast majority of the signals detected in those areas remain unidentified.

Infrared ion spectroscopy (IRIS) has recently gained increased recognition as a highly valuable MS-based tool for the identification of features detected in untargeted metabolomics experiments^10–14^. IRIS allows the generation of infrared (IR) spectra for a population of mass-selected ions inside the MS, therefore providing orthogonal information for any feature detected in the MS spectrum^14^. For instance, recent work in our group has demonstrated the identification and differentiation of saccharides^11,15^, drug metabolites^12^, prostaglandins^14^, amino acids^13^, and organic acids^14^ from various body fluids. Integration of untargeted metabolomics workflows based on LC-MS with feature identification using IRIS can therefore directly enable the discovery of biomarkers that would otherwise remain unidentified.

A previous metabolomics study from our group based on LC-MS alone produced multiple biomarkers for PKU^16^. Although LC-MS/MS analysis led to partial identification of several *m/z* features, their full molecular structures remained unclear. One such feature was identified as a Phe-hexose conjugate. However, from the fragmentation data, we were not able to provide direct information on the identity of the hexose and the exact structure of the conjugate, although a glucose-containing structure was tentatively assigned on the basis of the relatively abundant glucose blood levels. A recent study reports amino-acid galactose conjugates in dried blood spot analysis of galactosemia patients; however, these conjugates were also only analyzed using MS/MS^17^. In both studies, a direct N-linked structure was proposed.

Here, we use an integrated analytical approach including MS, nuclear magnetic resonance (NMR) spectroscopy, and IRIS to fully elucidate the molecular structure of the Phe-hexose conjugate. We report evidence that this conjugate is the result of an Amadori rearrangement to form an Amadori rearrangement product (ARP)^18^. ARPs are known intermediates in the formation of advanced glycation end-products (AGEs), which are well studied in, for example, diabetes mellitus^19,20^ but also implicated in complications of human aging in general^21^. Under conditions of diabetic hyperglycemia, AGEs arise from non-enzymatic glycation of proteins and are thought to contribute to, for example, vascular pathology by cross-linking extracellular matrix proteins. Our study shows that ARPs are also readily formed under conditions of aminoacidemia, which is not only relevant for PKU, but also for other IEMs for which amino acids accumulate. These findings suggest that ARPs form a general class of metabolites for inborn errors in amino-acid metabolism, which may function as biomarkers and contribute to insights into disease pathophysiology.

## Results

### LC-MS analysis of PKU plasmas

**Fig. 1:**
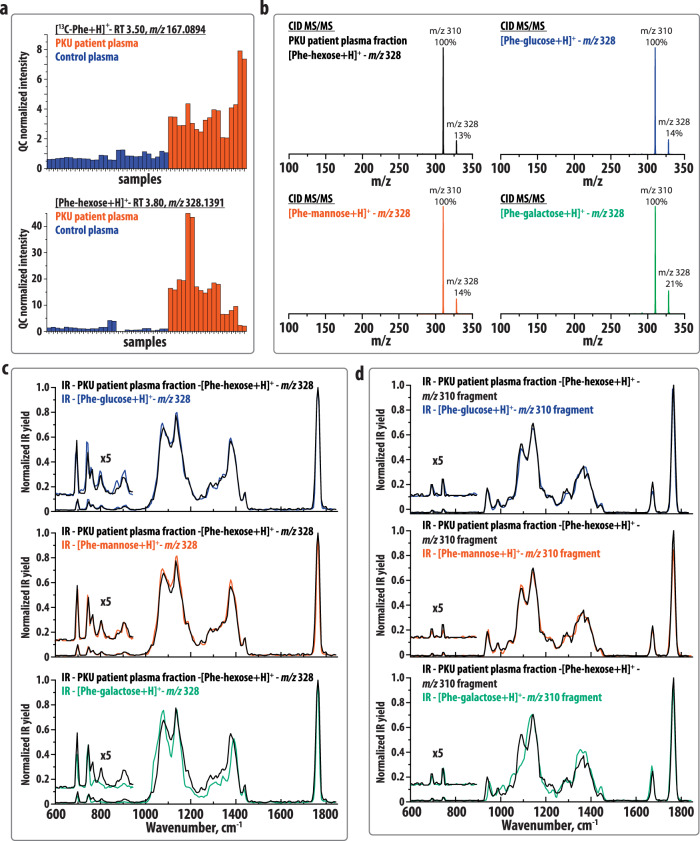
Overview of LC-MS and IRIS experiments. **a** Intensity plot of protonated ^13^C-Phe (*m/z* 167.0897) and the Phe-hexose (*m/z* 328.1391) observed in nine plasma samples of PKU patients (red) and 14 control plasmas (blue). Each sample was analyzed as two technical replicates, shown as consecutive bars in the figure. The ordering of samples is the same in both panels. The average fold changes (PKU patient/control) of Phe and the Phe-hexose are determined at 5 and 14, respectively. Comparison of **b** collision-induced dissociation (CID) MS/MS spectra and **c** IRIS spectra of the protonated ions ([M + H]^+^, *m/z* 328) of the Phe-hexose isolated from PKU patient plasma and the Phe-glucose, Phe-mannose, and Phe-galactose reference standards. **d** Comparison of the IRIS spectra of the water-loss fragments of the protonated ions ([M + H–18]^+^, *m/z* 310) of the Phe-hexose isolated from PKU patient plasma and the three reference standards.

We analyzed plasma samples of nine PKU patients using a validated untargeted LC-MS method previously described as next-generation metabolic screening^7^. Figure 1a plots the detected intensity of protonated ^13^C-Phe (*m/z* 167.0897 (+ESI)) and the previously reported Phe-hexose conjugate (*m/z* 328.1391 (+ESI)) in the plasma of controls (blue) and PKU patients (red) measured in duplicate. Both signals are found to be elevated in the plasma of PKU patients in comparison with controls and, as reported earlier^16^, the Phe-hexose shows a larger fold change than Phe (average fold change of 14 and 5, respectively). We note that Phe-hexose levels are not correlated to Phe- levels (see Fig. S1 for a correlation plot) likely owing to several factors, including plasma hexose levels. To identify the hexose involved in the formation of the Phe-hexose conjugates, we synthetically prepared reference standards for Phe-glucose and two closely related isomers, Phe-mannose, and Phe-galactose (see method section). Figure 1b compares collision-induced dissociation MS/MS spectra for the protonated ions of the three reference standards and the Phe-hexose from one of the patient samples. The four ions show highly similar fragmentation behavior, indicating that the LC-MS-feature is likely a Phe-hexose; however, it is impossible to distinguish the three hexose conjugates on this basis.

### Metabolite identification using IRIS

The Phe-hexose and the three reference standards were further analyzed using IRIS. Figure 1c shows a comparison of the IR spectrum obtained for the protonated Phe-hexose ion to spectra obtained for the protonated reference compounds. Here, it can be seen that the IR spectra of both the Phe-glucose as well as Phe-mannose ion are nearly identical to the Phe-hexose spectrum measured from the patient plasma. These results imply that the Phe-hexose metabolite results from the conjugation of either glucose or mannose with Phe, but also suggest that the Phe-glucose and Phe-mannose conjugation products are either structurally identical or are indistinguishable by their IR spectra. The Phe-galactose spectrum shows subtle but clear differences relative to the other spectra, especially in the region below 1000 cm^−1^, implying that the Phe-hexose metabolite in the body fluid of PKU patients is not the Phe-galactose conjugation product.

In order to further verify these results, IR spectra were obtained for the dehydrated MS/MS fragment ions ([M + H–18]^+^, *m/z* 310) produced by collision-induced dissociation of *m/z* 328. Figure 1d contains a comparison between the IR spectra of the mass-selected fragment ions. Again, the IR spectra derived from the Phe-glucose, Phe-mannose, and Phe-hexose (patient plasma) ions are highly similar, whereas the Phe-galactose-derived spectrum shows clear differences, here especially in the 900-1200 cm^−1^ region.

### Characterization of reference standards with NMR spectroscopy

**Fig. 2:**
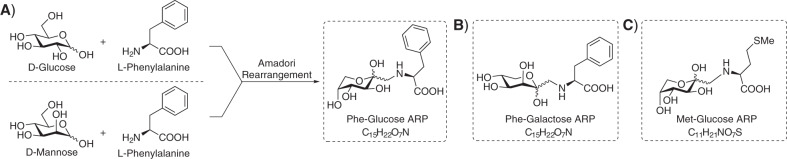
Chemical structures of the amino acid-hexose standards determined by NMR spectroscopy. **a** Reaction of d-glucose and d-mannose with l-phenylalanine results in the ARP product Phe-glucose ARP. **b** Structure of the Phe-galactose ARP reaction product. **c** Structure of the Met-glucose ARP reaction product. All structures are found to be Amadori rearrangement products (ARPs).

Although the abundance of the Phe-hexose ion in the PKU plasmas is too low to be characterized by NMR spectroscopy, we measured NMR spectra for the three reference standards in order to obtain more insight into the molecular structure of the products, resulting from the conjugation of Phe with each of the three hexoses. Figures S2 and S3 show the NMR spectra obtained for the Phe-glucose conjugate and the identified molecular structure is shown in Fig. 2a. Long-range couplings were observed between the hexose, the amino acid, and the bridging methylene, which shows that the synthesis product is a ketoamine. This can be formed from the Schiff base resulting from the reaction of the aldehyde group of a ring-opened sugar molecule and the amine group of an amino acid via the well-known Amadori rearrangement (see Scheme S1)^18^. Analysis of the ^1^H–^1^H coupling constants shows that the product adopts a ^4^C_1_ conformation.

Figures S4-S6 contain the NMR spectra obtained for the Phe-mannose and Phe-galactose conjugation products. A similar interpretation led to the conclusion that all three Phe-hexose conjugates undergo an Amadori rearrangement to produce ketoamines, also known as ARPs. Figure 2a, b shows the molecular structures assigned to the Phe-hexose reaction products. Here, we find that the Phe-mannose and Phe-glucose-ARPs are identical, directly in line with the IRIS results discussed above. This can be explained by the Amadori rearrangement reaction mechanism during which the stereochemistry at the C-2 position is scrambled (see Scheme S1).

Based on these results we conclude that the synthetic Phe-hexose standards are indeed ARPs and not an *N*,*O*-acetal as reported earlier based on multistage MS^n^ fragmentation^16^. The Phe- and hexose moieties are, in line with those results, connected via a bond between the hexose C-1 and Phe-amine group. Using the spectral similarity observed in Fig. 1, we conclude that the *m/z* 328 ion observed in the plasma samples of PKU patients is the ARP, resulting from the rearrangement of a Phe-glucose or Phe-mannose conjugate. This demonstrates the advantage of a molecular identification approach combining NMR spectroscopy and IRIS; where NMR spectroscopy gives precise structural information on the reference standards, IRIS matches the fingerprints of reference standards with low-abundance metabolites, which cannot be probed directly by NMR spectroscopy.

### IR spectral interpretation using quantum-chemical calculations

In order to identify the *m/z* 310 fragment ions and to provide an explanation for the spectral differences and similarities observed between the IR spectra shown in Fig. 1, we performed quantum-chemical calculations at the B3LYP/6-31++G(d,p) level of theory (see Fig. S7) that allow us to assign vibrational normal modes to the observed IR features. Comparing the IR spectra of the Phe-glucose and Phe-galactose ARPs (Fig. 1c) shows that distinctive spectral features are mainly in the region below 1000 cm^−1^. These features are attributed to a number of delocalized bending vibrations predicted in this region, which are likely affected by the stereochemistry at the hexose C-4 position. In the same region, both spectra contain two identical sharp peaks, which we attribute to out-of-plane CH-bending vibrations of the phenyl-group. These (localized) vibrations are barely affected by distant parts of the molecule^22^. The Phe-hexose fragment IR spectra (Fig. 1d) contain these vibrations as well, which explains the similarity of the fragment spectra in this region. As compared with the spectra of the precursors, the fragment IR spectra contain an additional peak ~1700 cm^−1^. This feature is attributed to a C=C stretching vibration, and its location provides evidence of OH-loss at the C-5 position and H-loss at the C-6 position of the hexose, yielding an ion with a double bond between C-5 and C-6. In addition, the fragment IR spectra contain two peaks just below 1000 cm^−1^ that can be attributed to bending vibrations of the dehydrated hexose moieties.

### ARP formation in other inborn errors of amino-acid metabolism

**Fig. 3:**
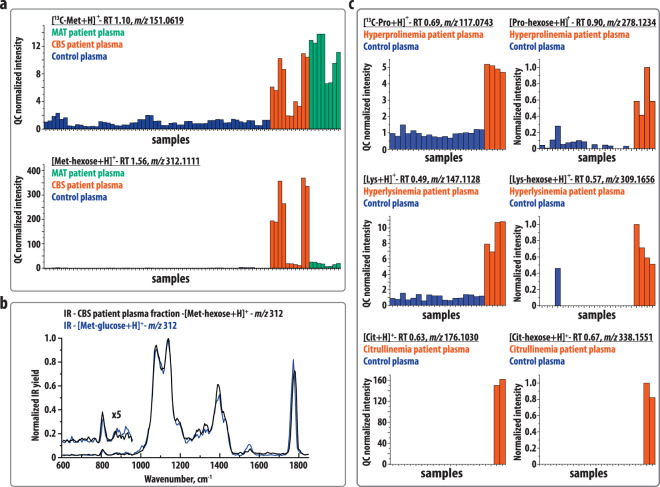
Results showing ARP formation from other accumulating amino acids. **a** Intensity plots of protonated methionine (Met) (*m/z* 150.0589), and the Met-hexose conjugate (*m/z* 312.1110) observed in plasma samples of methionine adenosyltransferase I/III deficiency (MAT) patients (green), cystathionine beta-synthase deficiency (CBS) patients (red), and control plasmas (blue). The average fold changes (patient/control) of Met and the Met-hexose are determined at 11 and 16 for MAT and 6 and 164 for CBS, respectively. **b** IRIS spectra of the protonated ion ([M + H]^+^, *m/z* 312) of the Met-hexose isolated from CBS patient plasma and the Met-glucose reference standards. **c** Intensity plots of protonated ^13^C-proline and lysine and their hexose conjugate observed in two plasma samples of patients (red) and 14 controls (blue), and of protonated citrulline and the citrulline-hexose conjugate observed in one plasma sample of a citrullinemia patient (red) and 17 controls (not visible on this scale). The ordering of samples is the same between the left and right panels. Most samples were analyzed as two technical replicates, shown as consecutive bars in the figures. A more-extended figure caption is available in Fig. S12.

In terms of chemistry, ARPs can in principle be formed non-enzymatically by the reaction of any amino acid with a hexose. It may, therefore, be expected that this reaction also takes place in the body fluids of patients with IEMs associated with elevated levels of other amino acids. We searched for ARP features in the data of previously analyzed plasma samples of patients with cystathionine beta-synthase deficiency (CBS) and methionine adenosyltransferase I/III deficiency (MAT) (both associated with high methionine (Met) levels). Figure 3a plots the detected intensity of protonated ^13^C-Met (*m/z* 151.0619 (+ESI)) and the Met-hexose conjugate (*m/z* 312.1111 (+ESI)) in five CBS patient plasmas (red) and four MAT patient plasmas (green) in comparison with control plasma samples (blue). The Met-signals are found to be elevated compared with controls in all MAT samples (average fold change of 11) and in all except one set of CBS samples (average fold change 6). The Met-hexose is elevated in all patient plasma samples (average fold change of 164 in CBS and 16 in MAT). The fold change is lower for MAT than for CBS, whereas the levels vary largely among CBS patient plasmas, which appears correlated to the levels of Met in the corresponding samples (see Figs. S8 and S9 for correlation plots). We identified the detected *m/z* 312 ion by recording its IR spectrum and that of a synthetically obtained Met-glucose ARP reference standard (see Figs. S10 and S11 for the NMR spectroscopy analysis and Fig. 2c for the identified structure of the reference), using the same approach as described above for the Phe-hexose conjugates in PKU. The spectral comparison in Fig. 3b clearly matches the *m/z* 312 ion to the Met-glucose ARP reference standard. This supports the hypothesis that amino acid-hexose conjugates are formed more generally in inborn errors of amino-acid metabolism and are not specific to PKU. We expanded this observation by looking for amino acid-hexose conjugates in the LC-MS data of previously analyzed plasma samples of patients with hyperprolinemia (type II, associated with high proline), hyperlysinemia (type I, associated with high lysine), and citrullinemia type I (associated with high citrulline). In each of these plasma samples, we observed high levels of both the amino acid and of the corresponding hexose conjugate (see Fig. 3c). As a secondary amine, proline cannot undergo the Amadori rearrangement via an imine structure, but instead is expected to form an iminium ion, which can rearrange to an enamine, which can then proceed to form the Amadori product. Lysine has two primary amines and can thus form two distinct Amadori products. Citrulline has one primary amine and one urea, however as the urea is likely not as reactive we expect only one Amadori product to form in this case.

Using the same procedure as described above for the Phe-hexose ARPs, quantum-chemical calculations were performed for the Met-glucose ARP (see Fig. S7). Comparison of the Phe-glucose and Met-glucose ARP IR spectra (Figs. 1c and 3b) shows that they are overall remarkably similar. This indicates that the dominant IR features are related to the common parts of the structure, whereas vibrational bands of the phenyl- versus S-CH_3_-groups are likely weaker and buried underneath these modes. Interestingly, the two glucose-ARPs share the spectral feature ~800 cm^−1^ and the double feature ~900 cm^−1^, which were deviating in the Phe-galactose spectrum. Furthermore, the modes assigned as out-of-plane CH-bending vibrations of the phenyl-group (~700 and 750 cm^−1^) are absent in the Met-glucose spectrum, which further confirms their band assignment.

## Discussion

In this work, we identified a class of amino-acid metabolites using an integrated analytical approach combining MS and NMR spectroscopy with IRIS. Combining these methods provides a previously unattainable insight into the molecular structure of features detected in untargeted LC-MS studies. NMR spectroscopy provides detailed molecular structure information for samples of high purity, here synthetically obtained reference compounds, where MS has the sensitivity to detect low-abundance metabolites but does not provide the required structural information. IRIS can now bridge the gap between both techniques, providing the direct information on molecular structure that has been lacking in metabolomics studies.

As the formation of ARPs is a process that can occur spontaneously, though slowly, under certain chemical conditions, we evaluated the extent of their formation under conditions of sample storage and preparation to evaluate their utility as reliable disease biomarkers. We observed a slowly increasing concentration of conjugation product as a function of years of storage when analyzing plasma and cerebrospinal fluid samples stored at −20 °C. In this study, we avoided deviations thereof by only including plasma samples that were analyzed within 3 years of collection. In our data analysis, ARP formation owing to storage could also be detected by the presence of ARPs formed from background non-elevated plasma amino acids that do form after long-term storage (−20 °C) but are not present in freshly collected samples. In addition, the formation of ARPs during sample preparation was ruled out as we did not find observable levels of the corresponding ARP in plasma samples spiked with l-phenyl-d5-alanine. Nevertheless, diagnostic routines using ARPs as biomarkers should avoid chemical conditions that induce spontaneous amino acid-hexose conjugation.

The Amadori rearrangement, as an intermediate in the Maillard reaction^23^, is well studied in the context of food chemistry,^24^ but also in the biochemical context of protein glycation^25,26^. In this process, free amino groups in proteins, most often on the sidechains of lysine and arginine residues, react with reducing sugars, especially when present in high concentrations such as in diabetes^20,26^. This reaction forms products that slowly rearrange to ARPs. These ARPs degrade further via several reaction pathways, yielding so-called AGEs^27^. AGEs are associated with the pathophysiology of diabetes^19,20^, but also have a role in neurodegenerative diseases such as Alzheimer’s, Parkinson’s, and Huntington’s disease^26,28^. Their putative pathophysiological effects occur upon the formation of crosslinks between glycated proteins, which then accumulate in tissues or bind to cell surface receptors, inducing oxidative stress and activation of inflammatory pathways. AGEs have also proven to be a target for therapy, for example, in the use of metformin as an AGE-inhibitor in diabetes type I and II^29,30^.

In this study, we show that ARPs are also readily formed from glucose and isolated amino acids, particularly, under conditions of aminoacidemia, which occur in several IEMs such as PKU, hyperprolinemia type I and II, citrullinemia type I, CBS-, and MAT deficiency. Our findings, therefore, indicate ARPs as a class of IEM-related metabolites, which could potentially be used as additional diagnostic biomarkers for aminoacidemia’s in parallel to amino-acid levels. Adding these biomarkers to diagnostic algorithms, such as used in neonatal screening, could increase their sensitivity, as our data suggest that the levels of ARPs maybe even more distinctive than the amino-acid levels themselves. In addition, ARPs may play a previously unrecognized role in IEM pathophysiology and open up new avenues to IEM therapy. Moreover, based on their chemical similarity to protein-derived ARPs, these compounds could potentially degrade to AGEs with their own pathophysiological effects. Further studies are needed to validate the clinical value of ARPs as biomarkers for inborn errors in amino-acid metabolism, and address the possible formation of AGEs from them.

## Methods

### Chemicals

Methanol, ethanol, water, and formic acid (LC-MS grade) used during the sample preparation procedure were obtained from Sigma-Aldrich (St. Louis, USA). Mobile phase solvents were prepared with LC-MS grade water and methanol obtained from Merck (Darmstadt, Germany) and LC-MS grade acetic acid from Fisher Scientific (Geel, Belgium).

### Synthesis

The corresponding sugar (1 eq.) and amino acid (1 eq.) were dissolved in MeOH (0.1 M). The mixture was refluxed for 1 h, after which the mixture was concentrated and applied to size-exclusion chromatography (P-2 Biogel). Fractions containing the product were lyophilized and obtained as a white solid.

### Sample preparation

Plasma samples were prepared following a procedure described previously^7^. Please refer to Table S1 for patient characteristics. The samples were thawed at 4 °C. In all, 400 μl of ice-cold methanol/ethanol 50:50 [v/v] was added to 100 μl of plasma and mixed using a vortex mixer for 15 s. For the samples used for metabolic profiling the ice-cold methanol/ethanol mixture contained five internal standards (caffeine-d3 0.88 μmol/L, hippuric-d5 acid 0.22 μmol/L, nicotinic-d4 acid 0.88 μmol/L, octanoyl-l-carnitine-d3 0.22 μmol/L, l-phenyl-d5-alanine 0.44 μmol/L (all from C/D/N Isotopes, Pointe-Claire, Canada)). Next, these dilutions were incubated for 20 min at 4 °C and subsequently centrifuged at 18,600 × *g* for 15 min at 4 °C. In all, 350 μl of the supernatant was transferred into new tubes and dried in a centrifugal vacuum evaporator (Eppendorf). The sample was reconstituted in 100 ml 0.1% formic acid in deionised water/methanol 90:10 [v/v], mixed using a vortex mixer for 15 s and centrifuged at 18,600 × *g* for 15 min at room temperature. In all, 90 μl of the supernatant was transferred to an autosampler vial and used for LC-MS analysis.

### LC-MS analysis

Metabolic profiling was performed as described previously^7^ using an Agilent 1290 ultra-high pressure LC system coupled to an 6545 QTOF mass spectrometer equipped with a dual ESI source. Data acquisition was done using Agilent MassHunter (version B.08.00). Positive mode separations were performed by injecting 2 µl on a Waters Acquity HSS T3 C18 column (100 × 2.1 mm i.d., 1.8 μm particles, 100 Å pore size) held at 40 °C using a mobile phase consisting of 0.1% [v/v] formic acid in water (mobile phase A) and 0.1 % [v/v] formic acid in 99:1 [v/v] methanol: water (mobile phase B). A flow-rate of 0.4 ml/min was used. After an initial time of 1 min at 99% A, a gradient was run to 100% B in 15 minutes followed by a hold at 100% B of 4 minutes. After a return to 100% A in 1 minute, an equilibration time of 4 minutes was used, leading to a total analysis time of 25 minutes.

Amino-acid and amino acid-hexose signals were extracted from the LC-MS data as peak areas using MassHunter Qualitative Analysis software Version B.08.00. In order to be able to combine data obtained in different LC-MS runs, resulting in signals were, where possible, normalized on the signal in a quality control (QC) plasma sample (normalized signal = signal/signal in QC sample). QC samples used in our laboratory consist of a mixture of 800 plasma samples collected from leftover material from the clinical chemistry laboratory. In several cases, the molecular signal was below the limit of detection in the QC sample. Here, signals were normalized on the highest signal obtained for this feature (normalized signal = signal/highest signal). Please refer to Supplementary Data 1 for the data underlying the presented histograms before and after normalization.

Fractions for the IRIS experiments were obtained using a Bruker Elute SP HPLC system and a Foxy R2 fraction collector equipped with two 96-well plates. These separations were performed with the same separation method as described above but a mobile phase consisting of 10 mM acetic acid in water (mobile phase A) and 10 mM acetic acid in methanol (mobile phase B) and an injection-volume of 15 µL.

### Infrared ion spectroscopy

IRIS experiments were performed in a quadrupole ion trap mass spectrometer (Bruker, AmaZon Speed ETD) modified for spectroscopy. Details of the hardware modifications and synchronization of the experiment with the infrared laser are described elsewhere^31^. The collected fractions and solutions of reference compounds (∼10^−7^ M in 50:50 methanol:water) were introduced at 120-180 μl/h flow rates to the electrospray source (+ESI). The ions of interest were mass-isolated and subjected to IR analysis. IR spectra were recorded using the FELIX infrared free-electron laser, which was set to produce IR radiation in the form of ∼10 μs macropulses of 50-150 mJ at a 10 Hz repetition rate (bandwidth ∼0.4% of the center frequency).

When the laser is resonant with a vibrational transition of the ions this leads to absorption of the IR photons, producing an increase in the ions’ internal energy and eventually leading to photodissociation. Thus, IR absorption can be observed by recording a fragmentation MS spectrum. IR spectra were constructed by plotting the fractional dissociation (IR yield = ΣI(fragment ions)/ΣI(parent+fragment ions)) as a function of IR laser frequency. The IR yield at each wavelength position of the laser was calculated from four to eight averaged fragmentation mass spectra. The IR frequency was calibrated using a grating spectrometer, and the IR yield was linearly corrected for frequency-dependent variations in the laser pulse energy^32^.

### NMR spectroscopy

^1^H and ^13^C NMR spectra were recorded on a Bruker 400 or 500 MHz spectrometer. Chemical shifts are reported in parts per million (ppm) relative to tetramethylsilane, or residual solvents as the internal standard. NMR data are presented as follows: chemical shift, multiplicity (*s* = singlet, *d* = doublet, *t* = triplet, *dd* = doublet of doublets, *m* = multiplet and/or multiple resonances), coupling constant (J) in Hertz (Hz), integration. All NMR signals were assigned on the basis of 1H NMR, 13C NMR, COSY, HSQC, HMBC, NOESY, and TOCSY experiments. NMR data are presented for the major anomer.

### Quantum-chemical calculations

Theoretical IR spectra were obtained by performing quantum-chemical calculations using the Gaussian16 software package^33^. Geometry optimizations and (harmonic) IR frequency calculations were performed using density functional theory at the B3LYP/6-31++G(d,p) level of theory. Input structures and conformers for the Phe-glucose fragment ion were generated based on chemical intuition. Input structures for the remainder of the calculations were generated using a previously described automated workflow for conformational searches based on the cheminformatics toolbox RDkit^13,34^. Using the SMILES structure format^35,36^ of the neutral molecule as input, this workflow generates all possible protonation isomers and performs a conformational search for each isomer using a distance geometry algorithm. This yields 500 conformers that are minimized using a classical force field (MMFF94)^37–41^ and clustered based on similarity resulting in a maximum of 10 conformers. These conformers are minimized at the semi-empirical PM6 level. Conformers and protonation isomers with relative energies <40 kJ/mol compared with the lowest-energy conformer were submitted for further optimization and IR frequency calculations at the B3LYP level of theory. Computational IR frequencies were scaled by 0.975 and convoluted with a Gaussian line shape function (20 cm^−1^ full widths at half maximum) to aid comparison with experimental spectra. Conformers were sorted on the basis of their relative free energy and, among the lowest-energy structures, the conformer was assigned, which showed the best match between the experimental and computational spectrum.

### Reporting summary

Further information on research design is available in the Nature Research Reporting Summary linked to this article.

## Supplementary information

Supplementary Information

Description of Additional Supplementary Files

Supplementary Data 1

Supplementary Data 2

Supplementary Data 3

Supplementary Data 4

Reporting Summary

## Data Availability

Supplementary Figures are available in the Supplementary Information file. Data underlying the histograms presented here are available in Supplementary Data 1. Data underlying the presented MS/MS spectra are available in Supplementary Data 2. Data underlying the experimental IR spectra are available in Supplementary Data 3. Data underlying the theoretical IR spectra and coordinates of the quantum-chemically optimized structures presented in Supplementary Fig. S7 are available in Supplementary Data 4. Raw research data files are archived locally on data servers and are available upon reasonable request from the corresponding authors (jonathan.martens@ru.nl and karlien.coene@radboudumc.nl).

## References

[1] Blau N, van Spronsen FJ, Levy HL (2010). Phenylketonuria. Lancet.

[2] Al Hafid N, Christodoulou J (2015). Phenylketonuria: a review of current and future treatments. Transl. Pediatr..

[3] van Spronsen FJ (2017). Key European guidelines for the diagnosis and management of patients with phenylketonuria. Lancet Diabetes Endocrinol..

[4] Leuzzi V, Chiarotti F, Nardecchia F, van Vliet D, van Spronsen FJ (2020). Predictability and inconsistencies of cognitive outcome in patients with phenylketonuria and personalised therapy: the challenge for the future guidelines. J. Med. Genet..

[5] Jahja R (2017). Cognitive profile and mental health in adult phenylketonuria: a PKU-COBESO study. Neurophyschology.

[6] Miller MJ (2015). Untargeted metabolomic analysis for the clinical screening of inborn errors of metabolism. J. Inherit. Metab. Dis..

[7] Coene KLM (2018). Next-generation metabolic screening: targeted and untargeted metabolomics for the diagnosis of inborn errors of metabolism in individual patients. J. Inherit. Metab. Dis..

[8] De Vijlder T (2018). A tutorial in small molecule identification via electrospray ionization-mass spectrometry: the practical art of structural elucidation. Mass Spectrom. Rev..

[9] Kind T, Fiehn O (2010). Advances in structure elucidation of small molecules using mass spectrometry. Bioanal. Rev..

[10] Martens J (2018). Unraveling the unknown areas of the human metabolome: the role of infrared ion spectroscopy. J. Inherit. Metab. Dis..

[11] Martens J (2017). Molecular identification in metabolomics using infrared ion spectroscopy. Sci. Rep..

[12] Martens J, Koppen V, Berden G, Cuyckens F, Oomens J (2017). Combined liquid chromatography-infrared ion spectroscopy for identification of regioisomeric drug metabolites. Anal. Chem..

[13] van Outersterp RE (2019). Reference-standard free metabolite identification using infrared ion spectroscopy. Int. J. Mass. Spectrom..

[14] Martens J (2020). Infrared ion spectroscopy: new opportunities for small-molecule identification in mass spectrometry - a tutorial perspective. Anal. Chim. Acta.

[15] Elferink H (2018). Direct experimental characterization of glycosyl cations by infrared ion spectroscopy. J. Am. Chem. Soc..

[16] Václavík J (2018). Structural elucidation of novel biomarkers of known metabolic disorders based on multistage fragmentation mass spectra. J. Inherit. Metab. Dis..

[17] DiBattista A (2017). Temporal signal pattern recognition in mass spectrometry: a method for rapid identification and accurate quantification of biomarkers for inborn errors of metabolism with quality assurance. Anal. Chem..

[18] Hodge JE (1955). The amadori rearrangement. Adv. Carbohydr. Chem..

[19] Yamagishi S-i, Nakamura N, Matsui T (2017). Glycation and cardiovascular disease in diabetes: a perspective on the concept of metabolic memory. J. Diabetes.

[20] Ruiz, H. H., Ramasamy, R. & Schmidt, A. M. Advanced glycation end products: building on the concept of the “common soil” in metabolic disease. *Endocrinology* **161**, bqz006 (2019). 10.1210/endocr/bqz006 PMC7188081 31638645

[21] Chaudhuri J (2018). The role of advanced glycation end products in aging and metabolic diseases: bridging association and causality. Cell Metab..

[22] van Outersterp, R. E. et al. Mass spectrometry-based identification of ortho-, meta-and paraisomers using infrared ion spectroscopy.* Analyst*, **145**, 6162–6170 (2020) 10.1039/d0an01119c 32924040

[23] Ellis GP (1959). The maillard reaction. Adv. Carbohydr. Chem..

[24] Silván JM, van de Lagemaat J, Olano A, del Castillo MD (2006). Analysis and biological properties of amino acid derivates formed by Maillard reaction in foods. J. Pharm. Biomed. Anal..

[25] Ulrich P, Cerami A (2001). Protein glycation, diabetes, and aging. Recent Prog. Horm. Res..

[26] Brownlee M (1995). Advanced protein glycosylation in diabetes and aging. Annu. Rev. Med..

[27] Vistoli G (2013). Advanced glycoxidation and lipoxidation end products (AGEs and ALEs): an overview of their mechanisms of formation. Free Radic. Res..

[28] Salahuddin P, Rabbani G, Khan RH (2014). The role of advanced glycation end products in various types of neurodegenerative disease: a therapeutic approach. Cell Mol. Biol. Lett..

[29] Ruggiero-Lopez D (1999). Reaction of metformin with dicarbonyl compounds. Possible implication in the inhibition of advanced glycation end product formation. Biochem. Pharmacol..

[30] Rahbar S (2000). Evidence that pioglitazone, metformin and pentoxifylline are inhibitors of glycation. Clin. Chim. Acta.

[31] Martens J, Berden G, Gebhardt CR, Oomens J (2016). Infrared ion spectroscopy in a modified quadrupole ion trap mass spectrometer at the FELIX free electron laser laboratory. Rev. Sci. Instrum..

[32] Berden G, Derksen M, Houthuijs KJ, Martens J, Oomens J (2019). An automatic variable laser attenuator for IRMPD spectroscopy and analysis of power-dependence in fragmentation spectra. Int. J. Mass. Spectrom..

[33] Frisch, M. et al. Gaussian 16, Revision A. 03, Gaussian. Inc. (Wallingford, CT, 2016).

[34] Landrum, G. RDKit: open-source cheminformatics. (2006).

[35] Weininger D (1988). SMILES, a chemical language and information system. 1. Introduction to methodology and encoding rules. J. Chem. Inf. Comput. Sci..

[36] Weininger D, Weininger A, Weininger JL (1989). SMILES. 2. Algorithm for generation of unique SMILES notation. J. Chem. Inf. Comput. Sci..

[37] Halgren TA (1996). Merck molecular force field. V. Extension of MMFF94 using experimental data, additional computational data, and empirical rules. J. Comput. Chem..

[38] Halgren TA (1996). Merck molecular force field. III. Molecular geometries and vibrational frequencies for MMFF94. J. Comput. Chem..

[39] Halgren TA (1996). Merck molecular force field. I. Basis, form, scope, parameterization, and performance of MMFF94. J. Comput. Chem..

[40] Halgren TA, Nachbar RB (1996). Merck molecular force field. IV. conformational energies and geometries for MMFF94. J. Comput. Chem..

[41] Tosco P, Stiefl N, Landrum G (2014). Bringing the MMFF force field to the RDKit: implementation and validation. J. Cheminform..

