# Supplementary material for: Amadori rearrangement products as potential biomarkers for inborn errors of amino-acid metabolism
Source: Commun Biol. 2021 Mar 19;4:367. doi: 10.1038/s42003-021-01909-5 (PMC7979741; doi:10.1038/s42003-021-01909-5)
Supplement: Supplementary file 1 — Supplementary Information [file 42003_2021_1909_MOESM1_ESM.pdf]

Supporting information:

Amadori rearrangement products as potential biomarkers for inborn errors of amino acid metabolism

Rianne E. van Outersterp[1], Sam J. Moons[2], Udo F.H. Engelke[3], Herman Bentlage[1], Tessa M.A. Peters[3], Arno van Rooij[3], Marleen C.D.G. Huigen[3], Siebolt de Boer[3], Ed van der Heeft[3], Leo A.J. Kluijtmans[3], Clara D.M. van Karnebeek[4], Ron A. Wevers[3], Giel Berden[1], Jos Oomens[1,5], Thomas J. Boltje[2], Karlien L.M. Coene[3*§], Jonathan Martens[1*§]

[1]*Radboud University, Institute for Molecules and Materials, FELIX Laboratory, Toernooiveld 7, 6525 ED, Nijmegen, the Netherlands*

[2]*Radboud University, Institute for Molecules and Materials, Synthetic Organic Chemistry, Heyendaalseweg 135, 6525 AJ, Nijmegen, The Netherlands*

[3]*Department of Laboratory Medicine, Translational Metabolic Laboratory, Radboud University Medical Center, Geert Grooteplein Zuid 10, 6525 GA Nijmegen, the Netherlands*

[4]*Department of Pediatrics, Radboud Centre for Mitochondrial Medicine, Radboud University Medical Centre, Geert Grooteplein Zuid 10, 6525 GA, Nijmegen, Netherlands.*

[5]*van't Hoff Institute for Molecular Sciences, University of Amsterdam, 1098XH Amsterdam, Science Park 908, The Netherlands*

*corresponding author

§ These authors jointly supervised this work

E-mail: jonathan.martens@ru.nl, karlien.coene@radboudumc.nl

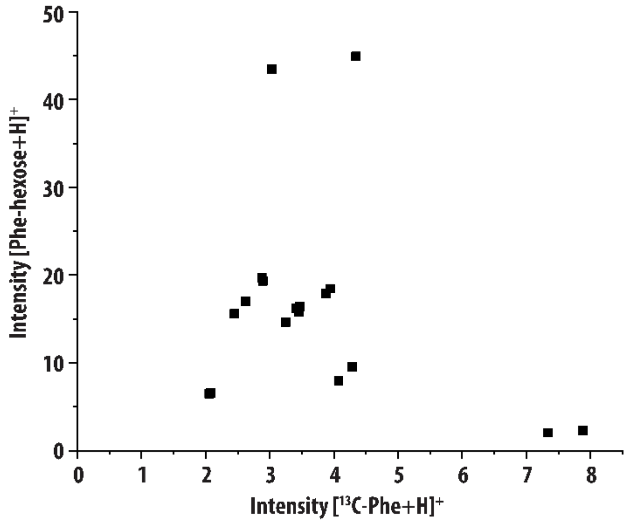

Figure S1 | Plot of the intensity of protonated Phe-hexose (m/z 328.1391) versus the intensity of protonated ^{13}C-Phe (*m/z* 167.0897) observed in nine plasma samples of PKU patients. All signals are normalized on the signal in the quality control sample.

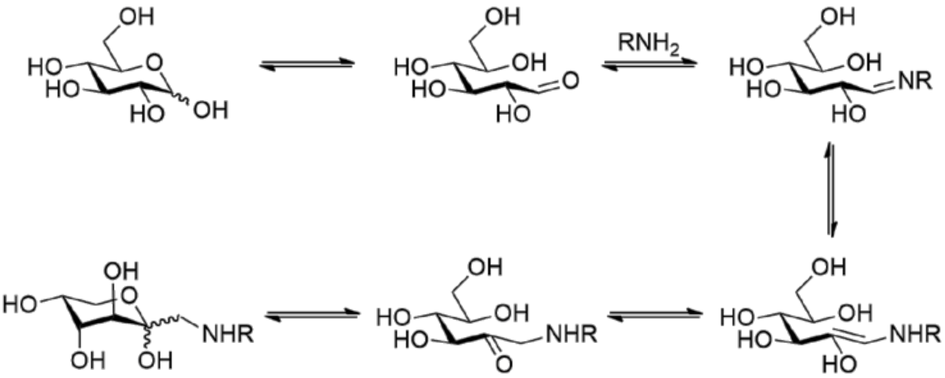

Scheme S1 | Mechanism of the conjugation of an amino acid (general structure RNH_2) with glucose and the following Amadori rearrangement transforming the amino acid-glucose conjugate into an Amadori rearrangement product (ARP), via the formation of a Schiff base.

a

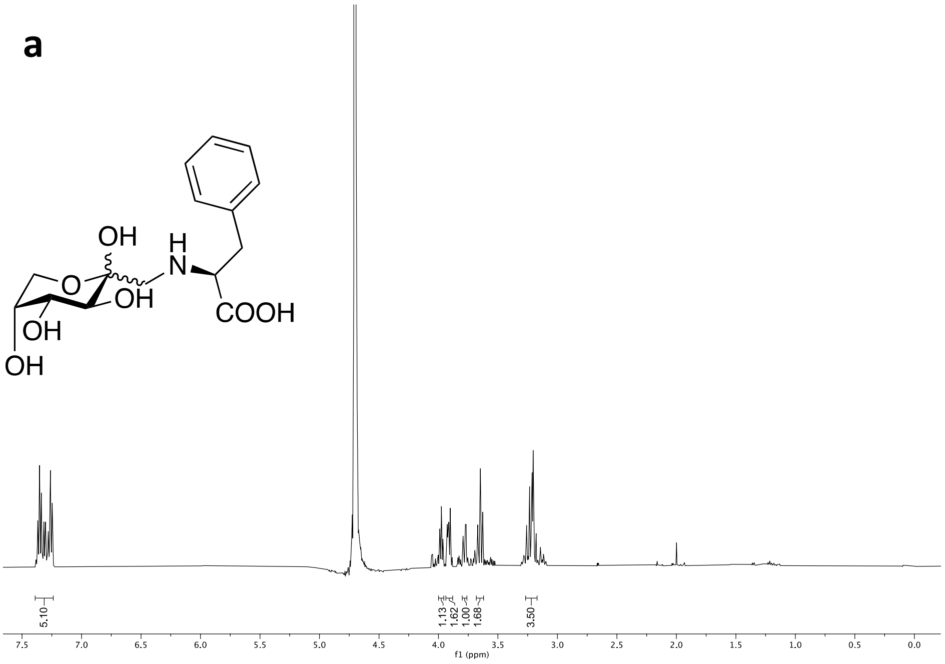

b

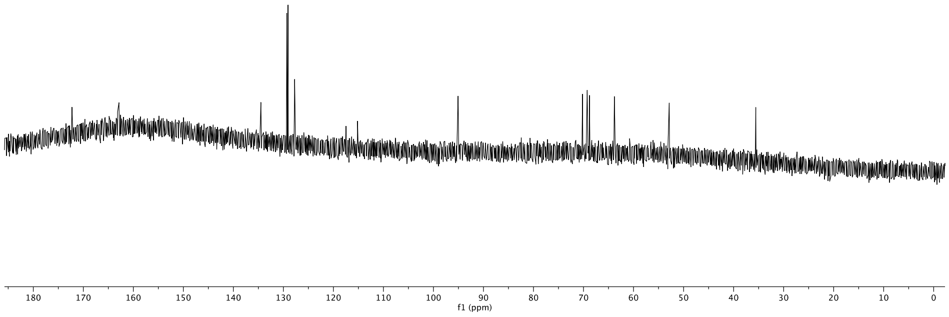

Figure S2 | ^{1}H NMR (a) and ^{13}C NMR (b) spectrum of the Phe-Glucose reference standard.
^{1}H NMR (500 MHz, D_2O) δ 7.47 – 7.32 (m, 6H), 4.06 (t, *J* = 6.5 Hz, 1H), 4.03 – 3.98 (m, 2H), 3.87 (dd, *J* = 9.9, 3.4 Hz, 1H), 3.77 – 3.71 (m, 2H), 3.36 – 3.24 (m, 4H). **^{13}C NMR** (126 MHz, D_2O) δ 172.25, 162.03, 129.32, 129.07, 127.75, 95.08, 70.19, 69.26, 68.81, 63.88, 63.83, 52.86, 35.93.

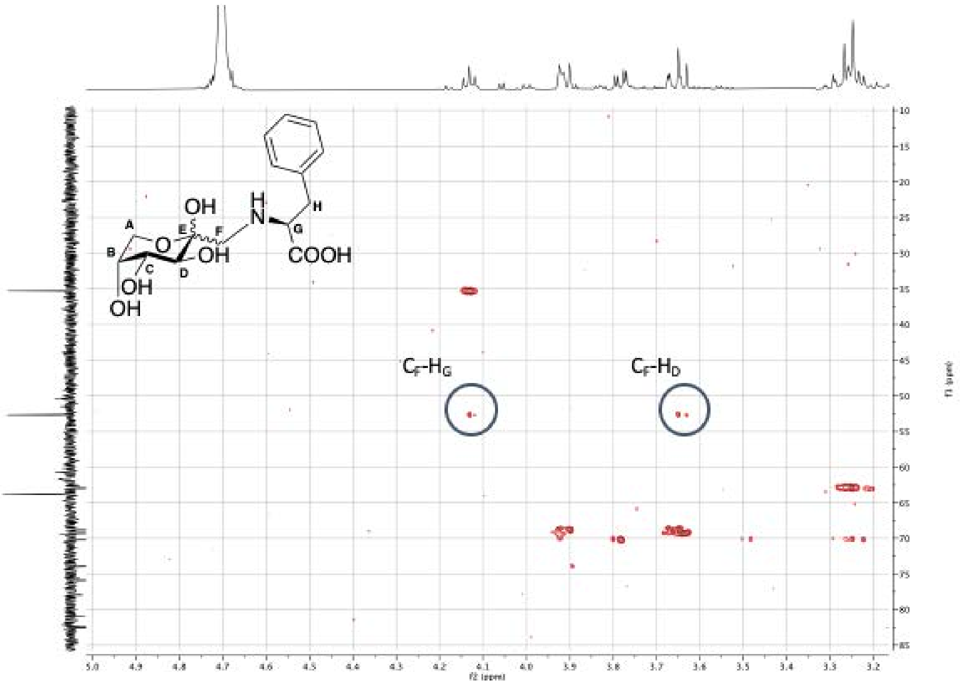

Figure S3 | ^{1}H-^{13}C HMBC of the Phe-Glucose reference standard. Long-range couplings are observed between the hexose (H_D), amino acid (H_G) and the bridging methylene (C_F)

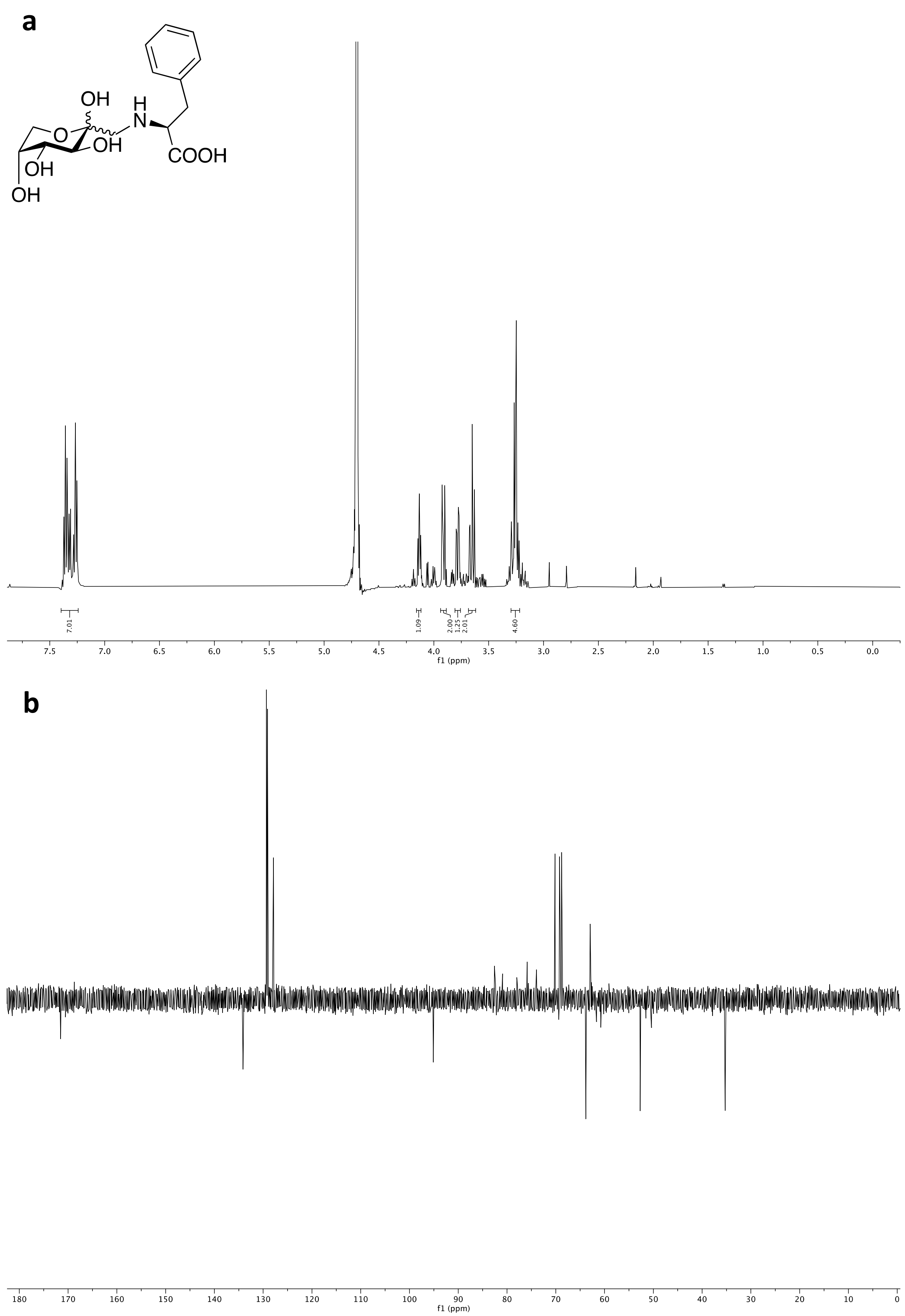

Figure S4 | ^{1}H NMR (a) and ^{13}C NMR (b) spectrum of the Phe-Mannose reference standard. The Phe-Mannose and Phe-glucose reference standards are found to be identical.

^{1}H NMR (500 MHz, D_2O) δ 7.39 – 7.30 (m, 3H), 7.27 (td, *J* = 8.1, 1.5 Hz, 2H), 4.13 (t, *J* = 6.5 Hz, 1H), 3.95 – 3.86 (m, 2H), 3.78 (dd, *J* = 9.8, 3.4 Hz, 1H), 3.69 – 3.61 (m, 2H), 3.31 – 3.18 (m, 4H). **^{13}C NMR** (126 MHz, D_2O) δ 171.53, 134.11, 129.34, 129.12, 127.88, 95.10, 70.16, 69.24, 68.81, 63.84, 62.96, 52.70, 35.28.

a

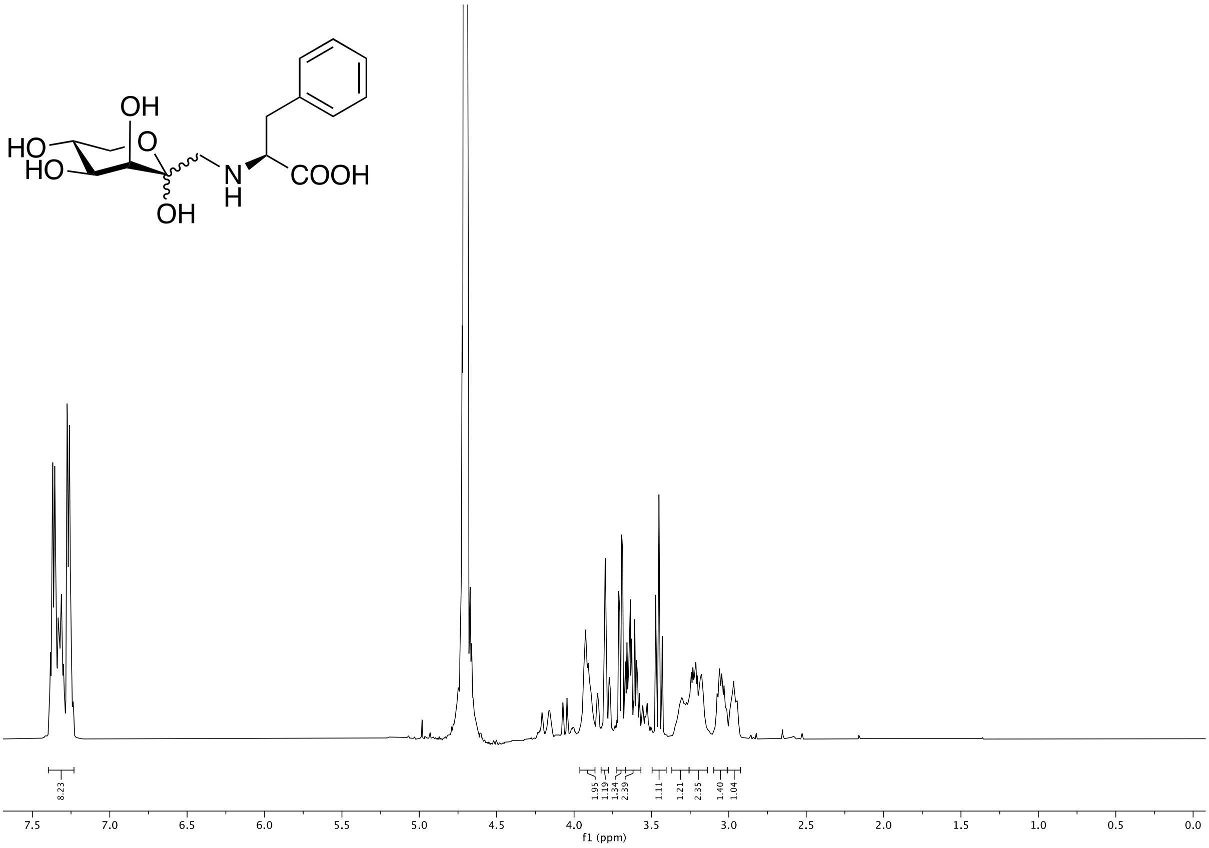

b

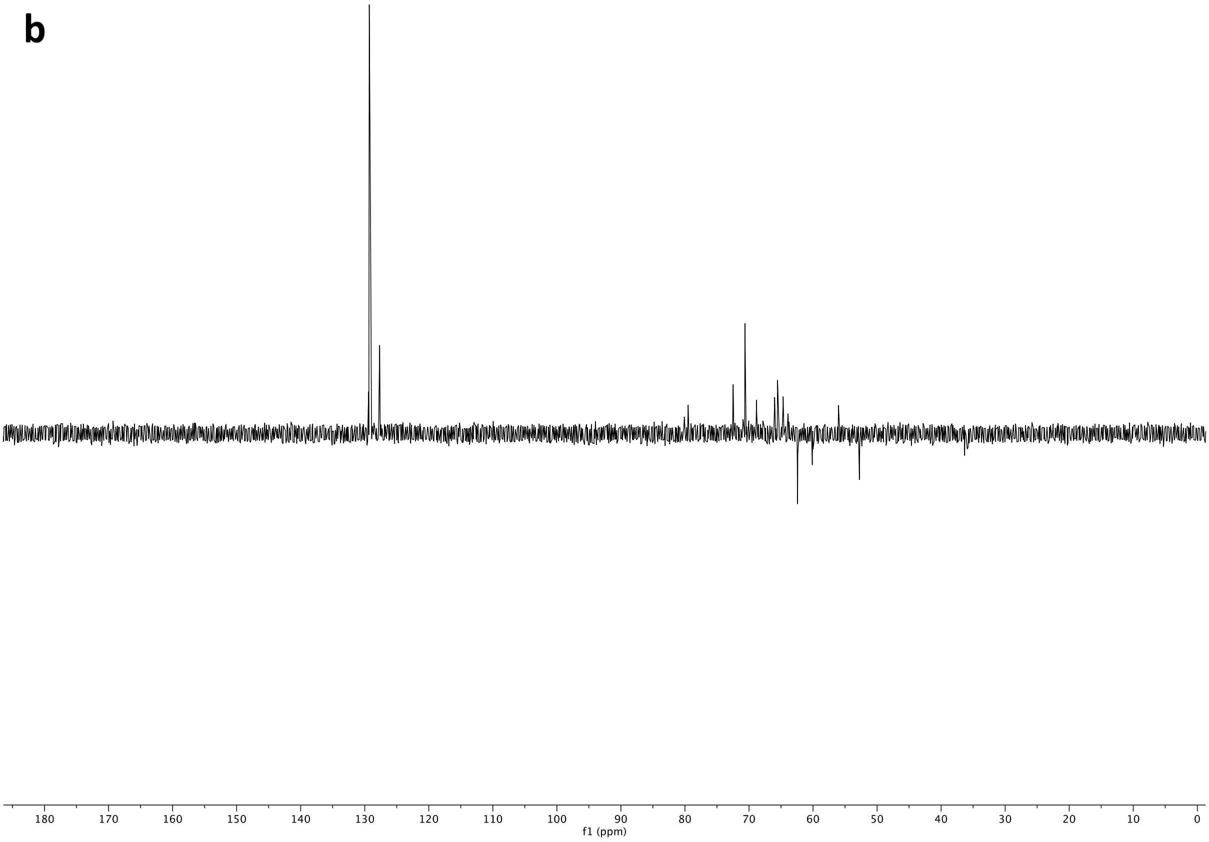

Figure S5 | ^{1}H NMR (a) and ^{13}C NMR (b) spectrum of the Phe-Galactose reference standard.
^{1}H NMR (500 MHz, D_2O) δ 7.42 – 7.13 (m, 5H), 3.97 – 3.87 (m, 1H), 3.78 (dt, *J* = 13.5, 2.2 Hz, 1H), 3.70 (m, 3H), 3.45 (t, *J* = 10.2 Hz, 1H), 3.36 – 3.12 (m, 2H), 3.05 (q, *J* = 7.9 Hz, 1H), 2.96 (d, *J* = 11.6 Hz, 1H). **^{13}C NMR** (126 MHz, D_2O) δ 171.03, 135.28, 129.29, 129.06, 127.66, 62.41, 60.12, 52.76, 36.33.

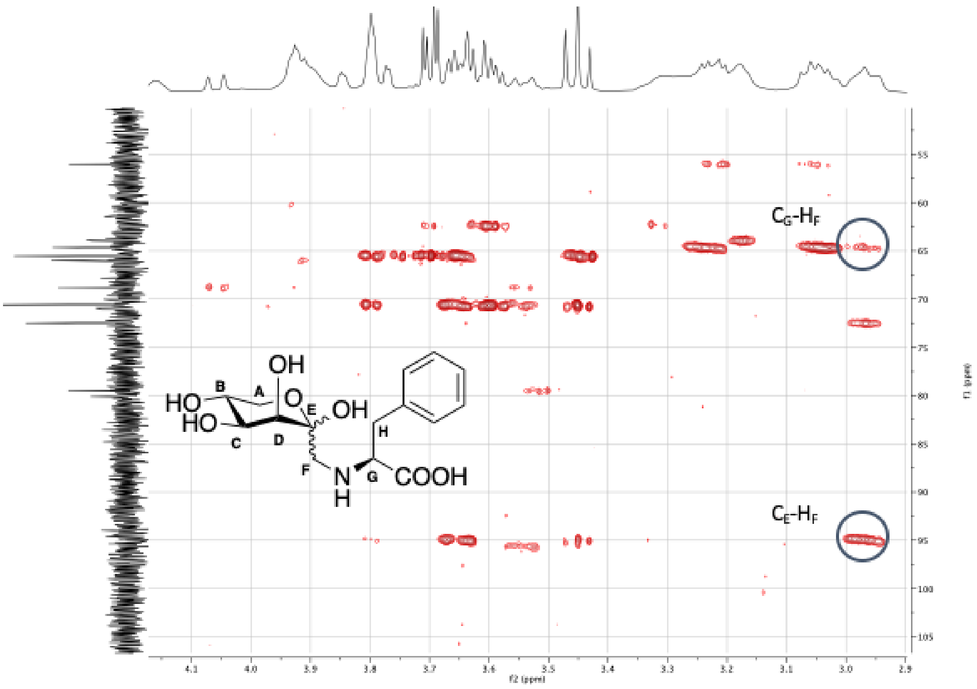

Figure S6 | ^{1}H-^{13}C HMBC of the Phe-Galactose reference standard. Long-range couplings are observed between the hexose (C_E), amino acid (C_G) and the bridging methylene (H_F)

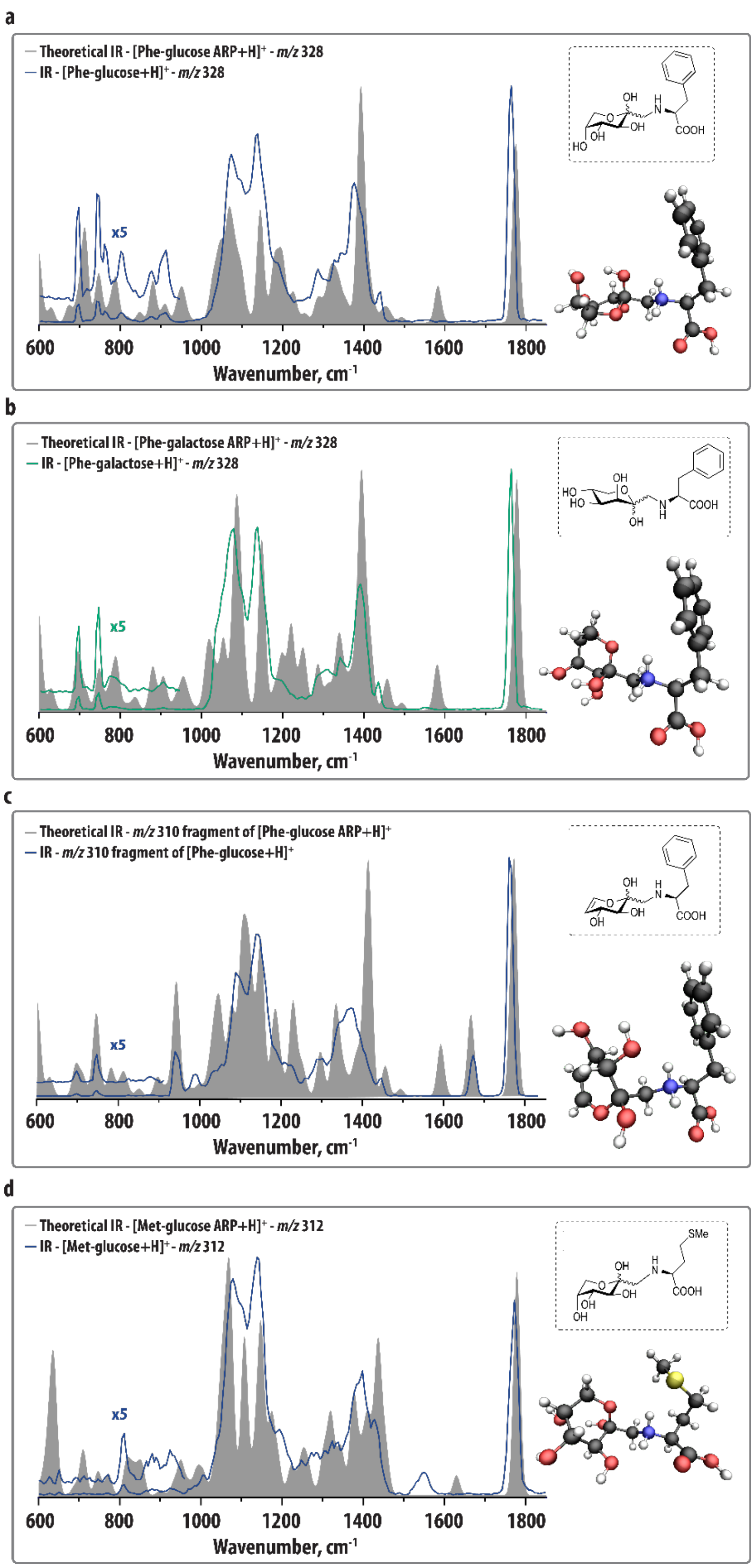

Figure S7 | Results of quantum-chemical computations. a-c) Comparison between IRIS spectra of the protonated ions of the **a)** Phe-glucose, **b)** Phe-galactose and **d)** Met-glucose reference standards and quantum-chemically computed IR spectra for each ion. **c)** Comparison between the IRIS spectrum of the *m/z* 310 fragment of the protonated ion of the Phe-glucose reference standard and its quantum-chemically computed IR spectrum. Structures of the reference compounds and DFT optimized structures are inlayed in each panel. The predicted peak around 1600 cm^{-1} is not observed for all Phe-hexose ions, which is likely due to it being too low in intensity to induce dissociation of the probed ions with the laser power used. This peak is not used for structural assignment. Each IR spectrum was normalized on the largest peak.

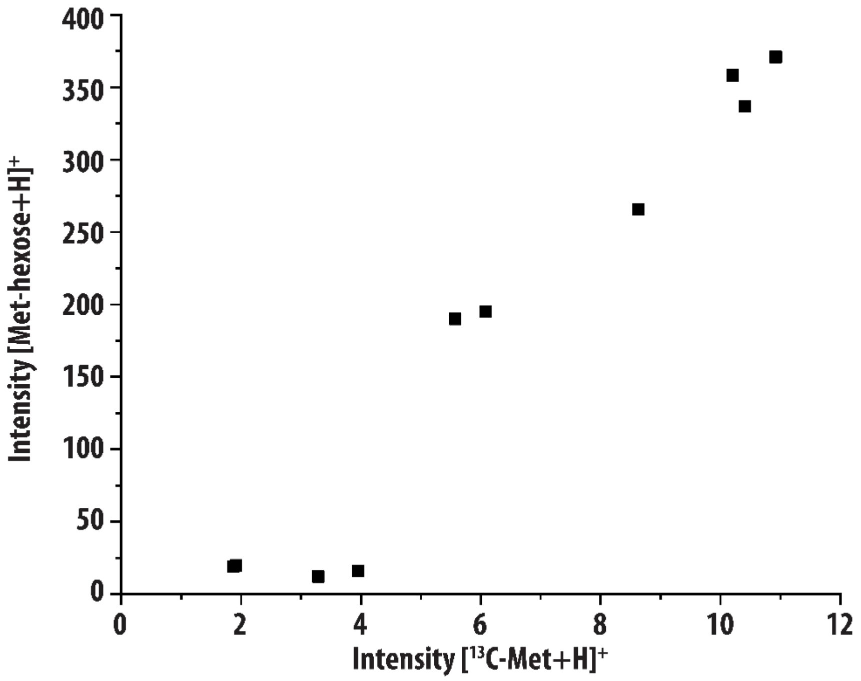

Figure S8 | Plot of the intensity of protonated Met-hexose (m/z 312.1111) versus the intensity of protonated ^{13}C-Met (*m/z* 151.0619) observed in plasma samples of CBS patients. All signals are normalized on the signal in the quality control sample.

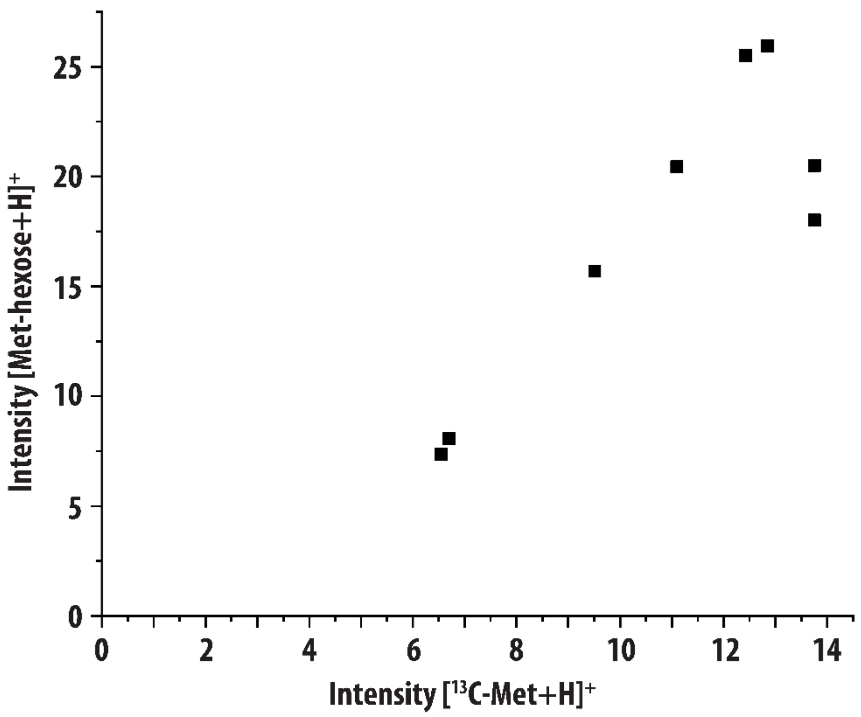

Figure S9 | Plot of the intensity of protonated Met-hexose (m/z 312.1111) versus the intensity of protonated ^{13}C-Met (*m/z* 151.0619) observed in plasma samples of MAT patients. All signals are normalized on the signal in the quality control sample.

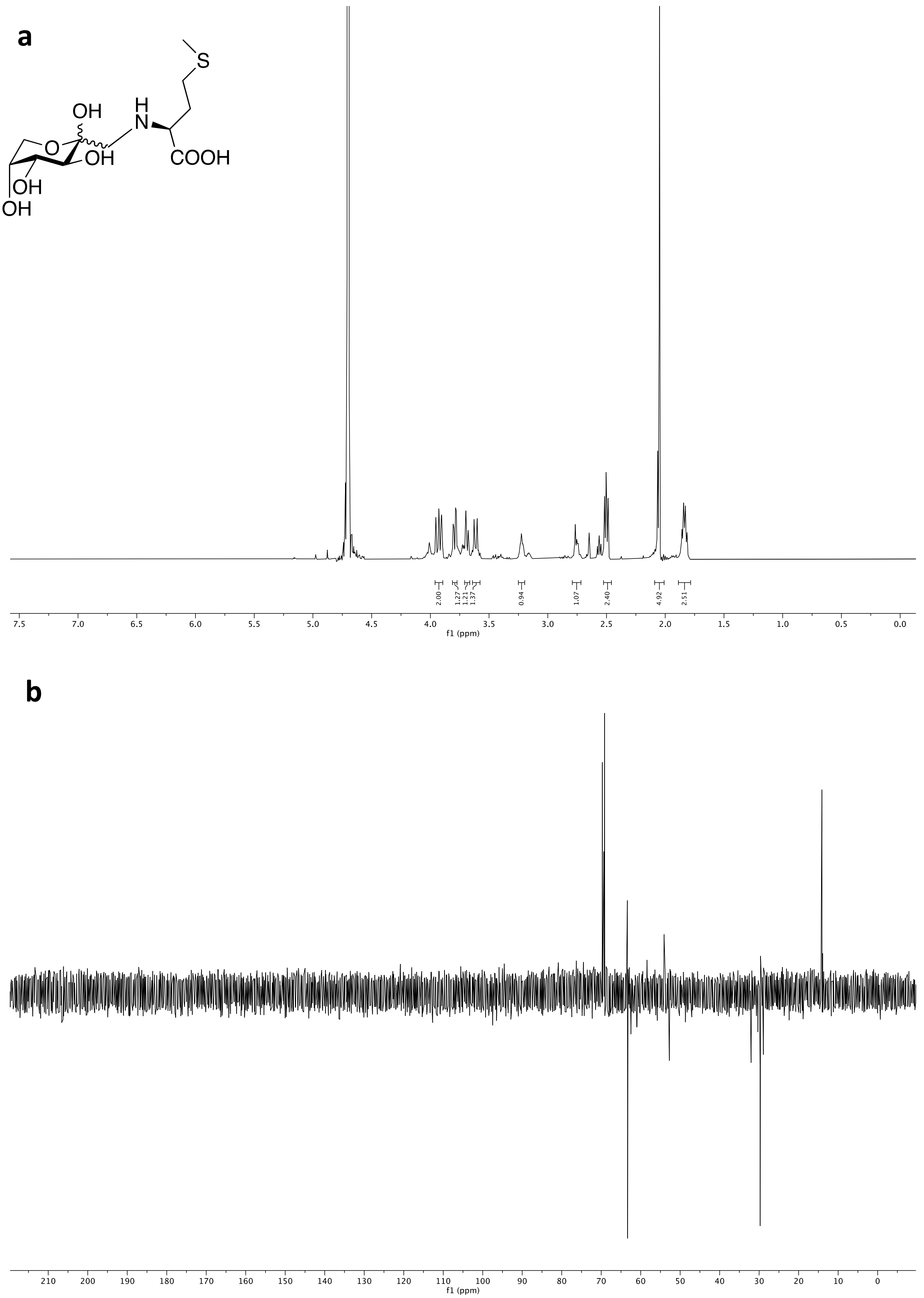

Figure S10 | ^{1}H NMR (a) and ^{13}C NMR (b) spectrum of the Met-Glucose reference standard. ^{1}H NMR (500 MHz, D_2O) δ 3.97 – 3.88 (m, 2H), 3.79 (dd, *J* = 10.0, 3.4 Hz, 1H), 3.72 – 3.66 (m, 1H), 3.61 (dd, *J* = 12.8, 1.9 Hz, 1H), 3.22 (d, *J* = 8.0 Hz, 1H), 2.80 – 2.72 (m, 1H), 2.50 (dd, *J* = 8.9, 6.4 Hz, 2H), 2.06 (d, *J* = 7.0 Hz, 3H), 1.84 (s, 2H). **^{13}C NMR** (126 MHz, D_2O) δ 97.56, 69.69, 69.31, 69.14, 63.37, 63.31, 52.75, 32.05, 29.74, 14.11.

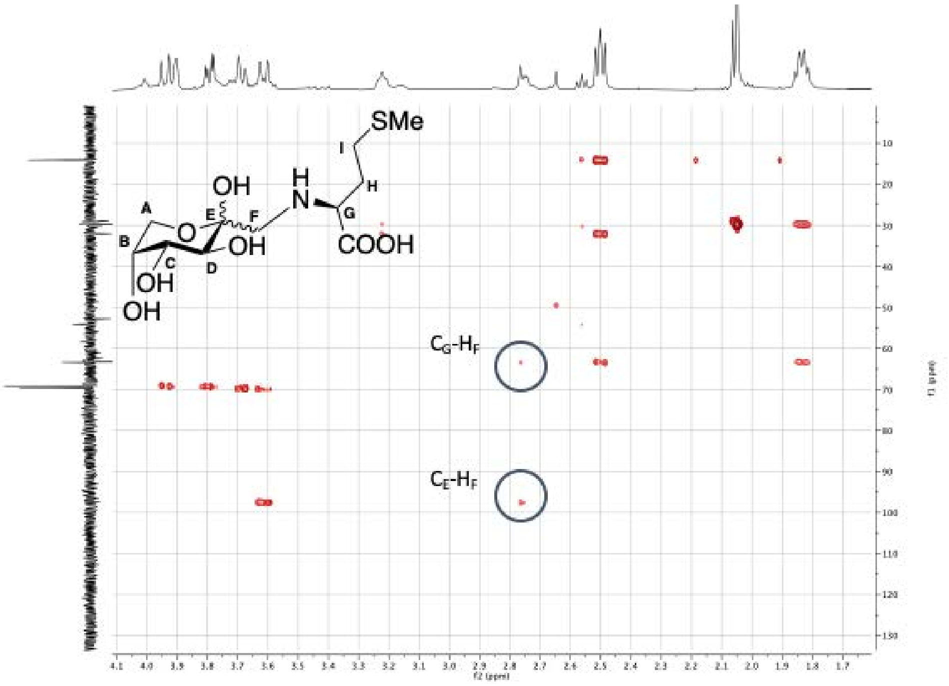

Figure S11 | ^{1}H-^{13}C HMBC of the Met-Glucose reference standard. Long-range couplings are observed between the hexose (E_D), amino acid (C_G) and the bridging methylene (H_F)

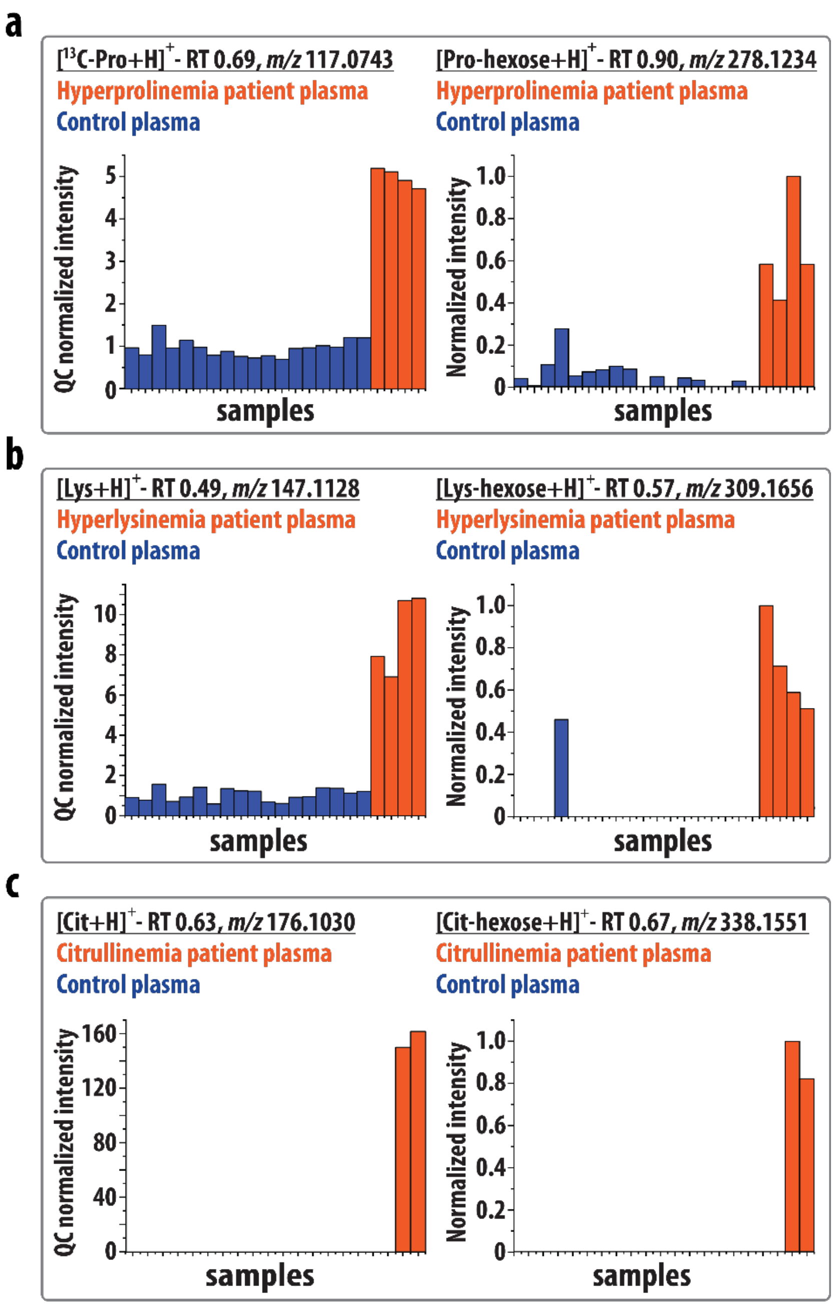

Figure S12 | (a) Intensity plots of protonated ^{13}C-proline (Pro, *m/z* 117.0743), and the Pro-hexose conjugate (*m/z* 278.1234) observed in two plasma samples of hyperprolinemia patients (red) and 14 controls. The average fold changes (patient/control) are 5 and 12, respectively. **(b)** Intensity plots of protonated lysine (Lys, *m/z* 147.1128) and the Lys-hexose conjugate (*m/z* 309.1656) observed in two plasma samples of hyperlysinemia patients (red) and 14 controls. The average fold changes (patient/control) are 9 and 28, respectively. In **(a)** and **(b)** the first ten bars (from left to right) represent controls which were analyzed as one technical replicate whereas the next 4 bars represent controls measured as two technical replicates (shown as consecutive bars in the figures). The ordering of samples is the same between left and right panels. **(c)** Intensity plot of protonated citrulline (Cit, *m/z* 176.1030), and the Cit-hexose conjugate (*m/z* 338.1551) observed in one plasma sample of a citrullinemia patient (red, two technical replicates) and 17 controls (blue, one technical replicate for 16 controls and two technical duplicate for one control). The average fold changes (patient/control) is 155 for Cit. The average fold change of the Cit-hexose could not be determined as it is not detected in any of the controls. The ordering of samples is the same in both panels.

Table S1 | Characteristics of patients corresponding to the studied samples. Samples are listed in the order in which they are presented in the manuscript.

Sample	Disorder	Amino acid level	Sex	Age
1	hyperphenylalanine/phenylketonuria	Phe 384 uM	Male	13 days
2	DHPR deficiency	Phe 355 uM	Female	7 months
3	hyperphenylalanine/phenylketonuria	Phe 528 uM	Female	10 years
4	hyperphenylalanine/phenylketonuria	Phe 241 uM	Female	19 years
5	hyperphenylalanine/phenylketonuria	Phe 359 uM	Female	6 months
6	classic phenylketonuria	Phe 1243 uM	Male	25 years
7	classic phenylketonuria	Phe 337 uM	Female	8 years
8	hyperphenylalanine/phenylketonuria, untreated	Phe 585 uM	Male	7 days
9	classic phenylketonuria, untreated	Phe 2232 uM	Male	7 days
10	cystathionine beta synthase deficiency	Met 383 uM	Male	59 years
11	cystathionine beta synthase deficiency	Met 591 uM	Male	21 years
12	cystathionine beta synthase deficiency	Not available	Male	57 years
13	cystathionine beta synthase deficiency	Met 117 uM	Female	22 years
14	cystathionine beta synthase deficiency	Met 674 uM	Female	Age unknown
15	methionine adenosyltransferase I/III deficiency	Met 469 uM	Female	4 years
16	methionine adenosyltransferase I/III deficiency	Met 503 uM	Female	4 years
17	methionine adenosyltransferase I/III deficiency	Met 172 uM	Female	1 month
18	methionine adenosyltransferase I/III deficiency	Met 464 uM	Male	1 year
19	Hyperprolinemia type II	Pro 2648 uM	Female	10 years
20	Hyperprolinemia type II	`Pro 1974 uM	Male	17 years
21	Hyperlysinemia type I	Lys 1006 uM	Male	3 years
22	Hyperlysinemia type I	Lys 1483 uM	Male	34 years
23	Citrullinemia type I	Cit 2951 uM	Male	4 years
