# Supplementary material for: Amadori rearrangement products as potential biomarkers for inborn errors of amino-acid metabolism
Source: Commun Biol. 2021 Mar 19;4:367. doi: 10.1038/s42003-021-01909-5 (PMC7979741; doi:10.1038/s42003-021-01909-5)
Supplement: Supplementary file 2 — Description of Additional Supplementary Files [file 42003_2021_1909_MOESM2_ESM.pdf]

Description of Additional Supplementary Files

File name: Supplementary Data 1

Description: LC-MS data underlying the histograms in Figures 1 and 3.

File name: Supplementary Data 2

Description: Data underlying the MS/MS spectra in Figure 1.

File name: Supplementary Data 3

Description: Data underlying the experimental IR spectra in Figure 1 and 3.

File name: Supplementary Data 4

Description: Data underlying the theoretical IR spectra and coordinates of the quantum-chemically optimized structures in Figure S6.
